# Magnetically Targeted Drug Transport Across a Tumor Cell Membrane Under Magnetic Field Gradients

**DOI:** 10.3390/ijms27115098

**Published:** 2026-06-04

**Authors:** Milan S. Kovačević, Relja Dragnić, Vladimir M. Marković, Ivona Kovačević, Daniele Tosi

**Affiliations:** 1 Department of Physics, Faculty of Science, University of Kragujevac, 34000 Kragujevac, Serbia; kovac@kg.ac.rs (M.S.K.); relja72@yahoo.com (R.D.); vmarkovic@kg.ac.rs (V.M.M.); 2 Faculty of Medicine, University of East Sarajevo, 73300 Foča, Bosnia and Herzegovina; 3 Faculty of Medicine, University of Belgrade, 11000 Belgrade, Serbia; ivkovacevic1911@gmail.com; 4 School of Engineering and Digital Sciences, Nazarbayev University, Astana 010000, Kazakhstan; 5 National Laboratory Astana, Laboratory of Biosensors and Bioinstruments, Astana 010000, Kazakhstan

**Keywords:** magnetic drug targeting, magnetophoretic drift, tumor cell membrane, transmembrane drug transport, magnetic Peclet number

## Abstract

Magnetic targeting of drug carriers is commonly studied at macroscopic scales, while its impact on drug transport across individual cell membranes remains poorly quantified. Here, we present a theoretical and numerical model of magnetically assisted drug transport across the membrane of a single tumor cell exposed to magnetic field gradients. Extracellular transport is described by an advection–diffusion equation that couples passive diffusion with magnetophoretic drift, whereas intracellular transport is governed by diffusion and first-order uptake kinetics. The cell membrane is modeled as a semi-permeable interface with finite permeability, providing explicit coupling between extracellular and intracellular domains. Assuming spherical symmetry, the coupled transport equations are solved using finite-difference schemes, with magnetic forcing represented through an effective drift velocity $v_{mag}$ and interpreted using the magnetic Peclet number. To enable a controlled comparison between healthy and tumor cells, identical geometric, diffusive, and magnetic parameters are used, while biological differences are introduced solely through membrane permeability and intracellular uptake rates. By separating cumulative membrane delivery from cumulative intracellular uptake, the model resolves ambiguities arising from heterogeneous uptake kinetics. The results show that magnetophoretic drift enhances near-membrane drug accumulation and effective transmembrane flux without modifying intrinsic membrane properties. Magnetic targeting therefore acts as a transport amplifier, magnifying pre-existing biological differences and producing a larger model-predicted delivery advantage in tumor cells. Overall, the framework identifies the magnetic Peclet number as the key parameter governing the transition from diffusion-dominated to drift-enhanced cellular drug transport.

## 1. Introduction

Achieving precise spatial control over drug distribution remains a major challenge in cancer therapy. Systemically administered anticancer drugs frequently fail to accumulate at sufficiently high concentrations within tumor tissue while simultaneously exposing healthy organs to toxic effects. This lack of selectivity often limits therapeutic efficacy and contributes to dose-limiting side effects [1,2,3]. Consequently, significant research efforts have been directed toward the development of advanced drug delivery platforms capable of enhancing localization and reducing off-target exposure.

Nanotechnology-based drug delivery systems offer unique opportunities to modulate drug transport, biodistribution, and release kinetics. Numerous nanocarrier architectures, including polymeric nanoparticles, lipid-based systems, dendrimers, nanogels, inorganic nanoparticles, and hybrid constructs, have been investigated as vehicles for anticancer agents [4,5,6,7]. Tumor localization of these carriers may be achieved through passive mechanisms such as enhanced vascular permeability, or through active targeting strategies that exploit molecular recognition or pathological features of the tumor microenvironment [8,9]. In addition, externally or internally triggered systems have been designed to release drugs in response to specific stimuli, including pH changes, enzymatic activity, hypoxia, temperature variations, and electromagnetic inputs [10,11,12]. However, the effectiveness of many biologically driven targeting approaches is compromised by the inherent heterogeneity of tumors and the partial overlap of biochemical signatures between malignant and healthy tissues [13,14]. Furthermore, the use of external energy sources such as light or ultrasound may be limited by tissue attenuation and unintended interactions with surrounding structures. In contrast, magnetic fields provide a noninvasive and bio-orthogonal means of interacting with engineered drug carriers, as they propagate through biological tissues with negligible absorption or distortion [15].

Magnetically responsive drug delivery systems typically incorporate superparamagnetic iron oxide nanoparticles (SPIONs), which exhibit strong magnetic susceptibility only under applied magnetic fields and minimal remanent magnetization upon field removal. SPIONs are typically composed of magnetite (Fe_3_O_4_) or maghemite (γ-Fe_2_O_3_), with core sizes generally in the range of a few to several tens of nanometers in order to maintain superparamagnetic behavior [16]. These characteristics enable dynamic control over particle motion while reducing aggregation-related risks [17]. When exposed to magnetic field gradients, SPION-loaded carriers experience directional forces that can bias their transport toward specific regions, thereby enhancing drug accumulation within tumors [18,19]. Magnetic actuation has also been utilized to induce localized heating or to trigger drug release from thermosensitive carriers [20,21].

It should be noted that the magnetic response of SPION-based carriers is not a fixed material property, but depends on several physicochemical factors, including the chemical composition of the magnetic core, the core size, and the relative contributions of magnetite (Fe_3_O_4_), maghemite (γ-Fe_2_O_3_), or mixed iron-oxide phases [16,22]. In biomedical applications, SPIONs are typically coated with biocompatible polymers, silica, dextran, or polyethylene glycol to improve colloidal stability, reduce protein adsorption, and prevent aggregation [16,23]. In the absence of appropriate surface functionalization, magnetic nanoparticles tend to agglomerate in physiological media, which significantly alters their hydrodynamic size, magnetic response, diffusion properties, and magnetophoretic mobility [23,24]. Therefore, the transport behavior of drug-loaded magnetic carriers should be interpreted in terms of effective physicochemical parameters rather than solely their nominal core properties.

While magnetic targeting has been extensively studied at the tissue and organ scales, its influence on drug transport at the cellular level remains less well characterized. Drug entry into tumor cells is governed by a combination of passive and active processes. While small molecules may cross the membrane by diffusion [25,26], nanoparticle-based drug carriers are predominantly internalized via active mechanisms such as endocytosis, including clathrin-mediated, caveolae-mediated, and macropinocytosis pathways [27,28]. In the present work, we adopt a continuum transport description in which transmembrane transfer is represented through an effective permeability term, enabling a quantitative coupling between extracellular accumulation and intracellular delivery without explicitly resolving specific biological uptake pathways. This effective description does not imply purely diffusive transmembrane transport of nanoparticles, but rather represents the net result of multiple underlying processes, including endocytosis, membrane binding, and intracellular trafficking, which are not explicitly resolved in the model.

In the presence of magnetic field gradients, drug-loaded magnetic carriers are subjected to additional forces that introduce a directed transport component, effectively coupling diffusion with magnetically induced drift [29]. This coupling may increase local drug concentration at the membrane interface and thereby enhance transmembrane flux.

Previous theoretical and numerical studies have suggested that magnetic forces can substantially modify nanoparticle trajectories, concentration profiles, and membrane interactions, leading to improved cellular uptake [30,31,32,33]. Nevertheless, a comprehensive framework linking magnetic field gradients to drug diffusion across tumor cell membranes is still lacking.

In this study, we examine magnetically targeted drug transport across a tumor cell membrane under applied magnetic field gradients. By integrating magnetic forces into a diffusion-based transport model, we analyze how magnetically induced drift alters concentration gradients and membrane transport dynamics. To obtain numerical solutions of the reaction–diffusion equations governing extracellular drug concentration and intracellular diffusion with uptake processes, we employ the finite difference method (FDM), a classical and widely validated technique for partial differential equations [25,34,35]. The approach consists of discretizing both the spatial domain and the temporal domain, replacing continuous derivatives with discrete approximations that can be implemented on a computational grid. The findings of this work aim to provide quantitative insight into the role of magnetic fields in enhancing cellular-level drug delivery and to support the rational design of magnetically guided therapeutic strategies.

Unlike previous models that primarily address magnetic drug targeting at tissue or vascular scales, the present study focuses on transport at the single-cell level, explicitly coupling extracellular magnetic drift with membrane-limited transport. By separating transmembrane flux from intracellular uptake, the model introduces the concept of magnetic transport amplification, providing a mechanistic explanation for enhanced drug delivery without altering intrinsic membrane properties.

## 2. Results and Discussion

Numerical simulations of magnetically targeted drug transport were performed using a coupled extracellular advection–diffusion and intracellular reaction–diffusion model, whose full formulation is provided in the Section 3. All simulations were carried out for a single spherical cell embedded in an extracellular medium, under the assumption of spherical symmetry. The parameter values were selected to represent physiologically realistic conditions for tumor cells and magnetically responsive drug carriers, while also enabling systematic exploration of diffusion-, membrane-, and magnetically dominated transport regimes.

Table 1 summarizes the physical, biological, and magnetic parameters used in the numerical simulations. These parameters define the representative healthy- and tumor-cell cases analyzed in this section, while the governing equations and numerical implementation are provided in the Section 3. The cell radius was fixed at R=10 μm, corresponding to a typical mammalian cell size, while the outer boundary of the extracellular domain was set to $R_{\infty }=20\;R$, sufficiently large to avoid artificial boundary effects during the simulated time intervals. Extracellular and intracellular diffusion coefficients were chosen in ranges consistent with reported values for nanoscale drug carriers and intracellular transport in the cytoplasm, respectively. In particular, the lower intracellular diffusivity reflects increased crowding and viscosity within the cellular interior. Magnetic nanoparticle properties, including particle volume, radius, and magnetic susceptibility, were selected to represent superparamagnetic iron oxide-based carriers commonly used in magnetic drug delivery applications. The surrounding medium was assumed to be weakly diamagnetic, consistent with aqueous physiological environments.

In the present simulations, magnetic forcing enters the transport equations through the product B dB/dr, which determines the magnetophoretic drift velocity $v_{mag}$. Therefore, changes in the magnetic field strength B and in the field gradient dB/dr affect the model through the same drift-velocity term. The range B=0.1–2 T is provided to indicate physically relevant magnetic field strengths for magnetic targeting configurations, whereas the numerical results are primarily interpreted through the resulting values of $v_{mag}$ and the magnetic Peclet number. In the parameter sweeps, the magnetic forcing was varied by changing the effective drift velocity, corresponding to different combinations of B and dB/dr. Increasing B from 0.1 to 2 T, at a fixed gradient, would increase $v_{mag}$ proportionally and would therefore produce the same qualitative effect as increasing the field gradient: stronger near-membrane accumulation, larger transmembrane flux, and enhanced intracellular uptake. Thus, the conclusions are governed by the magnitude of $v_{mag}$ or $Pe_{mag}$, rather than by B alone.

To provide an illustrative baseline for the spatiotemporal concentration fields, a representative parameter set was selected from the ranges listed in Table 1. The cell radius was set to R=10 μm, while the extracellular computational boundary was placed at $R_{\infty }=200\;\mu m$. The extracellular reference concentration was $c_{0}=1\;mol\;m^{-3}$, used as a normalization scale rather than as a prescribed physiological nanoparticle concentration.

The transport parameters were $D_{e}=1.0\times 10^{-11}\;m^{2}\;s^{-1}$, $D_{i}=5.0\times 10^{-13}\;m^{2}\;s^{-1}$, $P_{m}=5.0\times 10^{-7}\;m\;s^{-1}$, and $k_{u}=5.0\times 10^{-4}\;s^{-1}$. For the magnetic carrier, the particle radius, particle susceptibility, medium susceptibility, and vacuum permeability were set to $r_{p}=25\;nm$, $\chi_{p}=0.5$, $\chi_{m}=-9\times 10^{-6}$, and $\mu_{0}=4\pi \times 10^{-7}\;H\;m^{-1}$, respectively.

For the magnetic case, the applied field and field gradient were set to B=1 T and $dB/dr=50\;T\;m^{-1}$. The corresponding magnetic drift velocity was evaluated from Equation (4) using $\eta =1.0\times 10^{-3}\;Pa\;s$. To avoid overestimating radially convergent focusing in the simplified spherical model, the simulations use conservative effective drift velocities in the range $10^{-8}–10^{-6}\;m\;s^{-1}$, which represent hindered transport in the extracellular microenvironment.

The initial conditions were $c_{e}(r,0)=c_{0}$ for $R<r<R_{\infty }$ and $c_{i}(r,0)=0$ for 0<r<R. Simulations were performed over $t_{max}=50\;s$, which is comparable to or longer than the characteristic extracellular diffusive time scale for the selected parameters.

**Figure 1:**
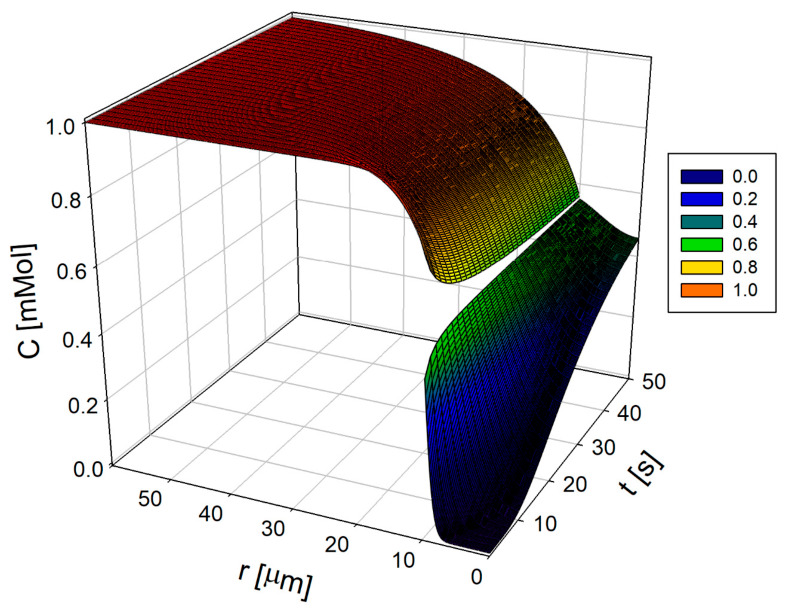
Three-dimensional mesh plot of the drug concentration c(r,t) displayed on a combined radial axis for the representative parameter set, in the absence of magnetic targeting ($v_{mag}=0$). The point r=0 corresponds to the center of the cell, while r=R=10 μm denotes the cell membrane separating the intracellular and extracellular domains.

**Figure 2:**
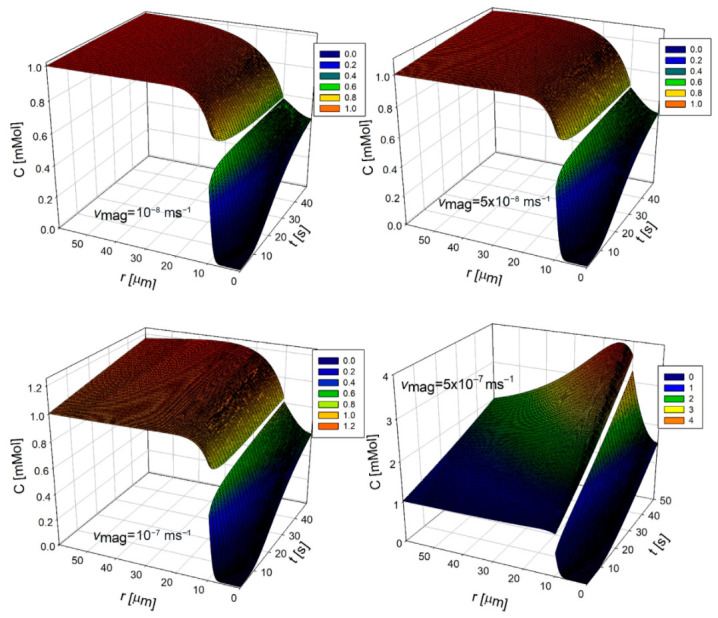
Three-dimensional mesh plot of the drug concentration c(r,t) displayed on a combined radial axis for the representative parameter set, with magnetic targeting included. The point r=0 corresponds to the center of the cell, while r=R=10 μm denotes the cell membrane separating the intracellular and extracellular domains. All other parameters are identical to those used in Figure 1.

For visualization, the intracellular and extracellular concentration fields were displayed on a single combined radial axis spanning 0≤r≤6R, rather than using separate plots for the two domains. The membrane location at r=R was indicated in the plots to identify the interface at which the permeability condition couples the two solutions. The plotted outer radius 6R was selected for clarity; beyond this range, the extracellular solution relaxes monotonically toward the far-field value $c_{0}$ as $r\to R_{\infty }$, and the larger numerical boundary $R_{\infty }=20R$ ensures that boundary effects do not contaminate the near-membrane dynamics during the simulated time window. The resulting mesh plots therefore emphasize the physically relevant near-cell region where magnetic drift modifies extracellular accumulation and, consequently, transmembrane delivery. Figure 1 presents the baseline no-field solution obtained by setting the magnetic drift term to zero, $v_{mag}=0$. Figure 2 shows the corresponding magnetically targeted cases for $v_{mag}=10^{-8}$–$5\times 10^{-7}\;m\;s^{-1}$, illustrating increased extracellular accumulation near the membrane and the associated enhancement of intracellular drug accumulation over time.

The numerical results clearly demonstrate that the introduction of a magnetic drift term fundamentally alters the spatiotemporal characteristics of drug transport. As shown in Figure 2, even moderate values of the magnetic drift velocity lead to a pronounced redistribution of concentration profiles, characterized by progressive accumulation at the cell membrane.

For the lowest investigated drift velocity, $v_{mag}=10^{-8}\;m\;s^{-1}$, transport remains predominantly diffusion controlled. The concentration profiles evolve smoothly in time, and no significant accumulation is observed at the membrane interface. In this regime, the magnetic contribution acts only as a weak perturbation and does not qualitatively modify the diffusion-driven dynamics. As the drift velocity increases to $v_{mag}=5\times 10^{-8}\;m\;s^{-1}$, deviations from purely diffusive behavior become evident. A noticeable steepening of concentration gradients near the membrane emerges, indicating the onset of magnetically assisted transport. The system enters a transitional regime in which diffusion and magnetic drift contribute comparably to the overall flux. A qualitatively different behavior is observed for $v_{mag}≳10^{-7}\;m\;s^{-1}$. In this case, magnetic drift dominates over diffusion in the vicinity of the membrane, leading to strong accumulation and the formation of a sharp concentration boundary layer. The membrane effectively acts as a bottleneck, where incoming magnetically driven flux cannot be instantaneously balanced by transmembrane transport. As a result, local concentrations significantly exceed bulk values, in some cases by more than an order of magnitude, as illustrated in the highest-drift scenario. Importantly, this accumulation is not an artifact of spherical symmetry, but a direct consequence of flux imbalance induced by the drift term. In physical terms, the magnetic field gradient generates a unidirectional transport component that continuously drives molecules toward the membrane, while the finite permeability limits their removal. The resulting concentration buildup is therefore a robust and generic feature of drift–diffusion systems with partially permeable boundaries. These results highlight the existence of a critical drift regime beyond which magnetic forces can no longer be treated as a small correction to diffusion. Instead, they fundamentally control transport kinetics and spatial organization. From an application standpoint, this suggests that magnetic targeting strategies may achieve substantial local drug enrichment at cellular membranes only when the magnetic drift velocity approaches or exceeds the characteristic diffusive velocity scale D/R. Below this threshold, magnetic effects remain negligible.

For the representative parameter set, the membrane Biot number is $Bi=P_{m}R/D_{e}=0.5$, indicating that membrane resistance and extracellular diffusive resistance are comparable in magnitude. Consequently, enhancements of the extracellular concentration in the near-membrane region can translate into an increased transmembrane flux, while the membrane still constitutes a non-negligible transport bottleneck. The diffusivity ratio $\gamma =D_{i}/D_{e}=0.05$ further confirms substantially slower spreading in the intracellular domain, consistent with cytoplasmic hindrance and delayed intracellular homogenization.

Based on the magnetic drift velocity considered at the upper end of the simulated range, $v_{mag}=5\times 10^{-7}\;m\;s^{-1}$, the corresponding magnetic Peclet number is ${Pe}_{m}=v_{mag}R/D_{e}\approx 0.5$. This value places extracellular transport in a moderately drift-enhanced regime, in which magnetic forcing is no longer negligible but diffusion still contributes substantially to mass transport. Importantly, this regime represents a realistic yet strongly biased transport scenario, avoiding the extreme upper-bound conditions associated with very large magnetic Peclet numbers. The drift velocities explored in Figure 2 therefore span a physically relevant range, illustrating how near-membrane accumulation and intracellular delivery efficiency increase progressively with magnetic forcing before reaching a membrane-limited regime.

Over the representative simulation window $t_{max}=50\;s$, the dimensionless extracellular diffusion time $\tilde{t}=D_{e}t_{max}/R^{2}\approx 2$ indicates that extracellular diffusion has sufficient time to establish substantial concentration gradients on the cellular length scale. In contrast, the intracellular uptake time scale $1/k_{u}\approx 2000\;s$ is much longer than $t_{max}$, implying that uptake acts primarily as a weak sink during the short-time simulations presented here. Its influence is therefore expected to become significant only in longer-time uptake dynamics or cumulative mass-transfer analyses.

**Figure 3:**
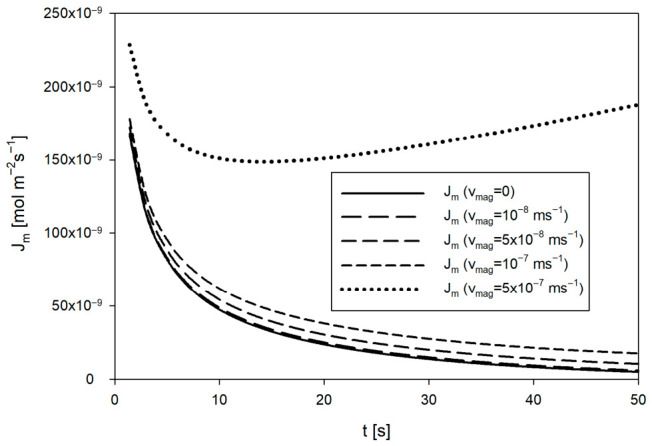
Time evolution of the transmembrane flux density $J_{m}(t)$ for different magnetic drift velocities $v_{mag}$. Magnetic drift enhances the initial flux by promoting transport toward the membrane but also accelerates the formation of near-membrane accumulation layers. At sufficiently large $v_{mag}$, the flux becomes limited by membrane permeability, leading to saturation and eventual decay despite increasing magnetic forcing.

Figure 3 presents the time evolution of the transmembrane flux density $J_{m}(t)$ for different values of the magnetic drift velocity $v_{mag}$. In the absence of magnetic drift ($v_{mag}=0$), the flux decreases monotonically with time. This behavior reflects the gradual relaxation of the concentration difference across the membrane as the drug enters the intracellular domain and the extracellular concentration near the membrane becomes depleted. Therefore, the no-field case represents a purely diffusion-controlled relaxation process without secondary flux enhancement.

When magnetic drift is introduced, the magnitude and temporal evolution of the transmembrane flux are modified. For moderate values of $v_{mag}$, magnetophoretic transport increases the near-membrane extracellular concentration, leading to larger values of $J_{m}(t)$ compared with the no-field case. For the highest investigated drift velocities, the flux may exhibit a non-monotonic time dependence, reflecting the competition between rapid near-membrane accumulation, membrane-limited transfer, and subsequent redistribution of the extracellular concentration field. This behavior is therefore associated with strong magnetic drift rather than with the purely diffusive no-field case.

Overall, these results demonstrate that magnetic drift enhances transmembrane flux most effectively during early times and for moderate drift velocities. Beyond this regime, the transport process becomes membrane-limited, and increasing magnetic forcing yields diminishing returns in terms of net uptake rate.

While the transmembrane flux density $J_{m}(t)$ directly quantifies the instantaneous transport rate across the membrane, it does not by itself provide a complete measure of intracellular delivery when intracellular uptake is present. In particular, the intracellular kinetics contains a first-order sink term, such that the intracellular free-drug pool can be depleted even if the membrane flux remains finite.

To quantify magnetic targeting efficiency in a dimensionless, time-resolved manner, we compare the intracellular amount obtained with magnetic drift $v_{mag}\neq 0$ to the corresponding baseline case without magnetic drift, $v_{mag}=0$. This factor directly measures the fold increase or decrease in the intracellular free-drug amount attributable to magnetic forcing. Because $M_{i}(0)=0$ in both cases, $\eta_{M}(t)$ is undefined exactly at t=0; in practice, it is evaluated for t>0, once $M_{i}(t;0)$ becomes nonzero.

**Figure 4:**
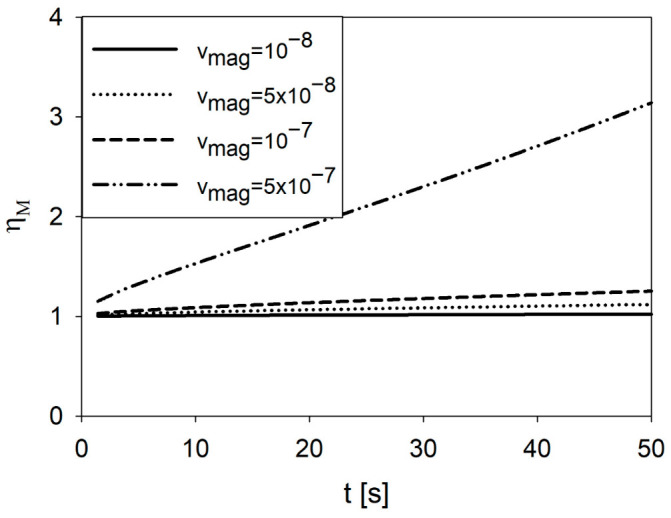
Time-dependent intracellular enhancement factor $\eta_{M}(t;v_{mag})=M_{i}(t;v_{mag})/M_{i}(t;0)$ for different magnetic drift velocities $v_{mag}$.

Figure 4 shows $\eta_{M}(t;v_{mag})$ for the same set of drift velocities used in the flux comparison. Consistent with the flux results, magnetic drift increases intracellular delivery at early times, reflected by $\eta_{M}(t)>1$ shortly after the onset of transport. However, unlike the instantaneous flux ratio, $\eta_{M}(t)$ integrates the delivery history while simultaneously reflecting uptake-induced depletion through the $-K_{u}M_{i}$ term. Consequently, $\eta_{M}(t)$ provides a more application-relevant measure of targeting performance, as it directly reports the net intracellular availability of a drug over time rather than the instantaneous membrane-crossing rate.

Figure 4 shows the time evolution of the intracellular enhancement factor $\eta_{M}(t)$ for four magnetic drift velocities. All curves exhibit an almost identical temporal shape and differ primarily in their growth rate, indicating that magnetic forcing modifies the magnitude of intracellular delivery rather than its qualitative time dependence. Over the simulated time interval, $\eta_{M}(t)$ increases approximately linearly with time, with no visible curvature or saturation. This behavior demonstrates that intracellular uptake does not yet compete with membrane delivery and confirms that the system operates in a short-time, delivery-dominated regime. The near-linear growth of all curves in Figure 4 motivates a common scaling form

(1) $$\eta_{M}(t;v_{mag})≃1+S(v_{mag})\;t,$$

where the slope $S(v_{mag})$ quantifies the magnetic-drift-induced enhancement of intracellular accumulation. A linear fit was applied to each curve, and the resulting slope values are summarized in Table 2.

The slope increases strongly and nonlinearly with magnetic drift velocity, reflecting the enhanced cumulative membrane influx induced by the magnetic field. Importantly, the temporal linearity observed for all cases implies that the uptake term has a negligible influence on the intracellular free-drug pool over the simulated time window. Uptake effects are therefore effectively factorized out in this regime, allowing magnetic targeting efficiency to be characterized by the single function $S(v_{mag})$. The dependence of S on $v_{mag}$ provides a compact summary of magnetic targeting performance. While the weakest drift velocity produces only a marginal enhancement over the baseline case, progressively stronger magnetic forcing leads to a rapidly increasing intracellular accumulation rate. This behavior mirrors the trends observed in the membrane flux analysis, confirming that magnetic drift enhances intracellular delivery primarily by increasing the cumulative membrane transport rather than altering intracellular kinetics. The enhancement rate increases with magnetic drift velocity, indicating stronger intracellular delivery under larger magnetophoretic forcing. Because the present analysis is based on a limited number of drift-velocity values, the dependence of $S(v_{mag})$ should be interpreted as an indicative trend rather than as a definitive scaling law. An exploratory fit to a power-law form,

(2) $$S(v_{mag})=A\;v_{mag}^{\beta },$$

yields β ≈ 1.3 over the investigated range. However, this exponent should be regarded as an approximate descriptor of the observed trend, since additional intermediate drift-velocity values would be required to establish the scaling behavior more conclusively. The main conclusion is therefore not the precise value of β, but the monotonic enhancement of intracellular delivery with increasing magnetic drift.

From the governing balance, saturation of $\eta_{M}(t)$ is expected only when intracellular uptake becomes comparable to membrane delivery, i.e., at times on the order of the uptake time scale. For the present parameters, this time scale lies well beyond the simulated interval, explaining the absence of saturation in Figure 4. At longer times, the linear growth is expected to transition into a plateau, with the asymptotic enhancement determined by the ratio of long-time averaged membrane fluxes. Taken together, Figure 3 and Figure 4 demonstrate that magnetic drift primarily enhances intracellular drug accumulation by increasing the cumulative membrane influx, while intracellular uptake does not influence the early-time dynamics. The function $S(v_{mag})$ therefore serves as a compact, quantitative measure of magnetic targeting efficiency and summarizes the effect of magnetic forcing on intracellular delivery in a single experimentally relevant parameter.

Furthermore, numerical simulations were performed for healthy and tumor cells using identical geometric, diffusive, and magnetic parameters, while allowing membrane permeability and intracellular uptake rates to differ, as described in the Section 3. This design ensures that any observed differences in drug accumulation arise solely from biological transport and processing properties rather than from magnetic forcing or geometric effects.

**Figure 5:**
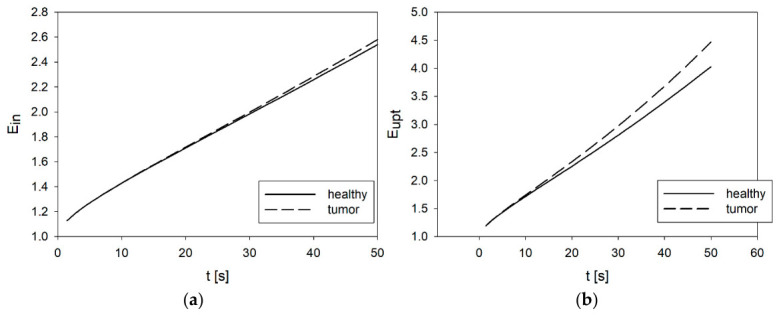
Time evolution of magnetic enhancement factors for healthy (solid line) and tumor (dashed line) cells: (**a**) enhancement of cumulative membrane delivery, $E_{in}(t)$; (**b**) enhancement of cumulative intracellular uptake, $E_{upt}(t).$ Tumor cells exhibit consistently larger enhancement, reflecting the combined effects of increased membrane permeability and higher intracellular uptake rates.

Figure 5 presents the time evolution of the magnetic enhancement factors for cumulative membrane delivery, $E_{in}(t)$, and cumulative intracellular uptake, $E_{upt}(t)$, for healthy and tumor cells. As shown in Figure 5a, the enhancement factor $E_{in}(t)$ increases approximately linearly with time for both cell types, reflecting the cumulative nature of magnetically assisted membrane transport. The tumor cell exhibits a consistently larger enhancement than the healthy cell, in agreement with the membrane-level analysis discussed above. This confirms that magnetic targeting more effectively increases total drug entry into tumor cells over time.

Figure 5b shows the corresponding enhancement of cumulative intracellular uptake, $E_{upt}(t)$. In this case, the difference between healthy and tumor cells is even more pronounced. While both curves increase monotonically, the tumor cell displays a substantially steeper growth, indicating that magnetic targeting produces a significantly larger increase in biologically effective intracellular drug processing in tumor cells. This amplification arises from the combined action of enhanced membrane delivery and higher intracellular uptake rates in tumor cells. Although a larger uptake rate reduces the instantaneous free intracellular drug pool, it simultaneously increases the cumulative amount of a drug irreversibly processed or retained inside the cell.

The distinction follows directly from the first-order uptake term used in the intracellular balance and from the definition of cumulative uptake introduced in the Section 3. The concentration $c_{i}(r,t)$ represents the freely diffusing intracellular drug pool, whereas the term $k_{u}c_{i}(r,t)$ represents the local rate at which this free drug is irreversibly bound, retained, metabolized, or otherwise removed from the freely diffusing pool. Therefore, $M_{upt}(t)$ accumulates the history of intracellular uptake over time, while $M_{i}(t)$ describes only the instantaneous amount of the free intracellular drug. As a result, a larger $k_{u}$ may reduce the instantaneous free intracellular concentration but increase the cumulative processed or retained amount, which is why both quantities must be interpreted separately.

**Figure 6:**
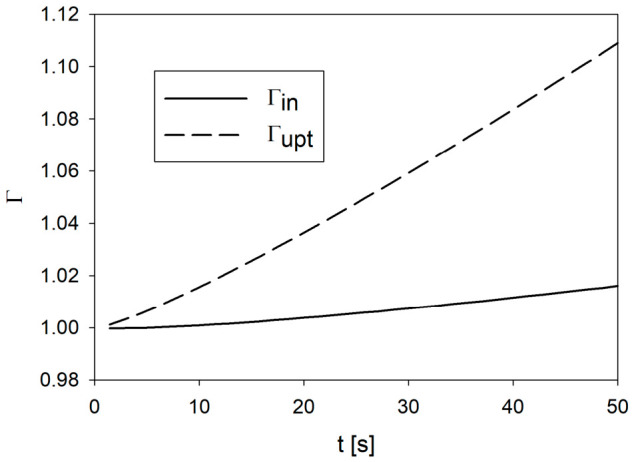
Relative therapeutic benefit of magnetic targeting expressed through the tumor-to-healthy enhancement ratios $\Gamma_{in}$ and $\Gamma_{upt}$. $\Gamma_{in}$ represents the relative benefit based on cumulative membrane delivery, whereas $\Gamma_{upt}$ represents the relative benefit based on cumulative intracellular uptake.

Figure 6 shows the time evolution of the relative therapeutic benefit of magnetic targeting, expressed through the tumor-to-healthy enhancement ratios $\Gamma_{in}$ and $\Gamma_{upt}$. The two curves therefore do not correspond to separate healthy and tumor cell responses, but to two different cumulative metrics: membrane delivery and intracellular uptake. $\Gamma_{in}$ quantifies the relative benefit based on cumulative membrane delivery, whereas $\Gamma_{upt}$ quantifies the relative benefit after intracellular uptake and retention are included. Values Γ>1 indicate that magnetic targeting produces a larger fractional enhancement in tumor cells than in healthy cells under identical magnetic field conditions.

Taken together, Figure 5 and Figure 6 demonstrate the importance of separating membrane-level delivery from intracellular drug fate when comparing healthy and tumor cells. Magnetic targeting enhances extracellular transport and near-membrane accumulation in the same physical manner for both cell types; however, the downstream biological response depends strongly on membrane permeability and intracellular uptake kinetics.

The cumulative membrane delivery $M_{in}(t)$ provides a clean, uptake-independent measure of magnetic targeting efficiency at the transport level, while the cumulative uptake $M_{upt}(t)$ captures the biologically relevant intracellular processing of the drug. The consistently larger values of $E_{in}$ and $E_{upt}$ for tumor cells, together with $\Gamma_{in}>1$ and $\Gamma_{upt}>1$, indicate that magnetic targeting preferentially benefits tumor cells by exploiting their increased membrane permeability and enhanced intracellular retention. These results show that magnetic targeting acts as a transport amplifier, magnifying intrinsic biological differences between healthy and tumor cells rather than creating them. Consequently, even moderate magnetic field gradients can produce a disproportionately larger therapeutic benefit in tumor cells under otherwise identical conditions.

The transition between diffusion-dominated and drift-enhanced regimes can be interpreted through the magnetic Peclet number, indicating that magnetic effects become significant only when directed transport competes with diffusive spreading.

An additional limitation of the present model is the simplified treatment of nanoparticle functionalization. In realistic biological environments, the transport behavior of magnetic nanoparticles strongly depends on surface coatings and functionalization strategies, which influence hydrodynamic size, colloidal stability, protein adsorption (opsonization), and interactions with immune cells [36,37]. In particular, nanoparticles lacking biocompatible polymer coatings are prone to rapid clearance by macrophages, which can significantly reduce their effective transport and targeting efficiency [37]. In the present framework, these effects are not modeled explicitly but are incorporated only implicitly through effective transport parameters, such as the hydrodynamic radius and the effective viscosity. A more detailed description of functionalization-dependent transport would require multiscale modeling approaches that explicitly resolve particle–biological interactions, which is beyond the scope of the current study.

Particle aggregation in biological media is also strongly influenced by the local medium composition. Ionic strength, pH, serum protein adsorption, and the formation of a protein corona can modify the surface charge and steric stabilization of magnetic carriers, thereby changing their colloidal stability, effective hydrodynamic size, diffusion coefficient, and magnetophoretic mobility [38,39]. In the present model, these effects are not treated as separate dynamic variables, but are included phenomenologically through effective carrier parameters such as the hydrodynamic radius, diffusion coefficient, and magnetic susceptibility.

It should be emphasized that aggregation effects were not separated from diffusion-driven behavior as an independent dynamic process in the present simulations. Instead, possible aggregation-related changes are represented through effective transport and magnetic parameters. In particular, aggregation or clustering would be expected to increase the effective hydrodynamic size, reduce the apparent diffusion coefficient, and modify the effective magnetic susceptibility and magnetophoretic mobility of the carrier system. Thus, the model distinguishes diffusion-driven behavior from aggregation effects at the parameter level rather than by explicitly resolving aggregate formation or breakup. A direct separation of diffusion, aggregation kinetics, and magnetically induced clustering would require an additional population-balance or particle-interaction model, which is beyond the scope of the current continuum framework.

Although the present study is formulated primarily for SPION-based drug carriers, the same modeling framework may be adapted to other magnetic nanomaterials. Alternative systems, including cobalt ferrite, manganese ferrite, nickel ferrite, and metallic or alloy-based magnetic nanoparticles, may provide higher magnetization, stronger magnetic anisotropy, or modified magnetophoretic response compared with conventional iron oxide carriers [16,23]. However, these potential magnetic advantages must be balanced against possible drawbacks, such as remanent magnetization, aggregation tendency, long-term biocompatibility, and toxicity concerns. SPIONs are used as the reference carrier class in the present study because they combine magnetic responsiveness with negligible remanent magnetization, established biomedical use, and tunable surface functionalization [16,40]. In the model, the specific material choice enters through effective quantities such as hydrodynamic radius, magnetic susceptibility, diffusion coefficient, and permeability-related transport parameters. Therefore, the framework can be extended to alternative magnetic nanomaterials by modifying these effective parameters, provided that their colloidal stability and biological compatibility are appropriately characterized.

Particle size represents an important limitation for tumor accumulation and tissue penetration. In general, nanoparticles larger than approximately 200 nm may exhibit reduced passive extravasation through leaky tumor vasculature and limited penetration into the tumor interstitium [37,41]. Magnetic targeting may partially compensate for this limitation by increasing the local residence time and concentration of magnetic carriers near the target region, thereby enhancing vascular retention, near-wall accumulation, and subsequent delivery to tumor tissue [22]. However, magnetic targeting should not be interpreted as completely overcoming size-dependent biological barriers. For larger carriers, the benefit of magnetic guidance is expected to depend strongly on vascular permeability, field-gradient strength, carrier magnetization, hydrodynamic size, and extracellular matrix resistance. In the present model, these effects are represented at the single-cell level through effective transport parameters, while explicit vascular extravasation and interstitial penetration are beyond the current scope.

A related limitation concerns the interpretation of larger carrier systems listed in Table 3. These entries are included to indicate representative magnetic carrier architectures and their possible magnetic response, but not all of them are expected to cross the plasma membrane as intact particles. For nanoscale carriers, cellular entry may occur through endocytic pathways, whereas larger microcarriers or nano-in-microparticle systems are more likely to act as extracellular or membrane-associated reservoirs that release the drug or smaller carrier components near the cell surface [27,37]. Therefore, in the present model, transmembrane transport should be interpreted as the effective transfer of the drug or releasable drug fraction into the intracellular domain, rather than as direct permeation of all magnetic carrier structures across the membrane. Explicit modeling of size-dependent carrier internalization, membrane wrapping, endosomal uptake, or carrier disassembly is beyond the scope of the current continuum formulation.

In the absence of drug loading, magnetic carriers would still undergo diffusion and magnetophoretic drift according to their hydrodynamic size, magnetic susceptibility, surface properties, and medium viscosity. However, they would not contribute to the drug concentration fields $c_{e}(r,t)$ and $c_{i}(r,t)$, nor to the cumulative delivery and uptake metrics defined in the present model. Therefore, drug-free carriers would represent a carrier-transport problem rather than a drug-delivery problem within the present framework. The uptake efficiency of the carriers themselves is not explicitly calculated here, because the model follows the effective concentration of a drug associated with magnetic carriers rather than the number density of internalized particles. In tumor cells, carrier uptake would depend on particle size, surface chemistry, protein corona formation, membrane interactions, and endocytic pathways [27,37]. These processes are represented only phenomenologically through effective permeability and uptake parameters, while a direct calculation of carrier internalization efficiency would require an additional particle number or receptor-mediated uptake model.

A further limitation of the present model is that particle aggregation, coating-dependent colloidal stability, and surface functionalization are not explicitly resolved. These factors are known to affect the hydrodynamic radius, diffusion coefficient, magnetic susceptibility, cellular interactions, and magnetophoretic mobility of SPION-based carriers [16,23]. In the present continuum formulation, their influence is represented through effective parameters such as $D_{e}$, $r_{p}$, $\chi_{p}$, and $v_{mag}$. This approach is suitable for identifying transport regimes and estimating the role of magnetic drift at the cellular scale, but it does not replace particle-level models or experimental characterization of a specific nanoparticle formulation.

It should be emphasized that the present model adopts an effective continuum description of transmembrane transport, in which the permeability term represents the net rate of drug transfer into the cell. This formulation does not imply that nanoparticles cross the membrane via passive diffusion. Instead, it provides a phenomenological representation of the combined effects of multiple biological processes, including endocytosis, membrane binding, vesicular trafficking, and intracellular release mechanisms [27,36]. While these processes are not explicitly resolved at the mechanistic level, their overall impact is incorporated into the effective transport parameters. This approach enables a tractable and quantitative analysis of magnetically enhanced delivery while maintaining consistency with known cellular uptake mechanisms.

A possible extension of the model would be to introduce a time- or coverage-dependent membrane permeability, $P_{m}(t)$, in order to account for carrier accumulation, membrane-associated retention, or partial blocking of available membrane transport sites.

The theoretical predictions of the present model could be experimentally validated using advanced magnetic characterization and imaging techniques. In particular, electron spin resonance (ESR/EPR) spectroscopy and imaging provide sensitive tools for probing the diffusion behavior, magnetic response, and cellular internalization of iron oxide nanoparticles in biological environments [42]. In addition, magnetic particle spectroscopy (MPS) enables quantitative characterization of nanoparticle dynamics and magnetic properties under oscillating magnetic fields, offering a direct means to assess magnetically induced transport processes [43]. These techniques provide complementary approaches for validating the predicted coupling between magnetic field gradients, nanoparticle transport, and cellular uptake, and have been increasingly applied in the context of magnetic drug targeting.

The present model is based on several simplifying assumptions that should be considered when interpreting the results. In particular, nanoparticle transport is described using effective continuum parameters, without explicitly resolving particle–particle interactions, functionalization-dependent behavior, or detailed cellular uptake mechanisms such as endocytosis. In addition, the model assumes spherical symmetry and homogeneous transport properties within each domain, while magnetic field effects are incorporated through a local gradient approximation.

Future developments of the model may include more detailed descriptions of nanoparticle functionalization, explicit coupling to intracellular trafficking pathways, and extension to heterogeneous tissue environments or multicellular systems. Such extensions would enable a more comprehensive and quantitatively predictive framework for magnetically targeted drug delivery.

## 3. Materials and Methods

The theoretical framework used to obtain the results presented above is based on a single-cell model in which a cell is represented as a sphere of radius R, embedded in an extracellular medium that extends to a finite outer radius $R_{\infty }$. Owing to the assumed spherical symmetry of the system, transport processes are described using a one-dimensional radial coordinate r. The computational domain is thus divided into two regions: the intracellular domain $\Omega_{i}=\{0\leq r\leq R\}$, corresponding to the interior of the cell, and the extracellular domain $\Omega_{e}=\{R\leq r\leq R_{\infty }\}$, representing the surrounding fluid environment in which drug-loaded magnetic nanoparticles are suspended. The model describes the spatiotemporal evolution of two concentration fields. The intracellular drug concentration is denoted by $c_{i}(r,t)$, while $c_{e}(r,t)$ represents the extracellular concentration of the drug carried by magnetic nanoparticles. These two fields are coupled through transport across the cell membrane located at r=R.

Magnetic targeting is modeled by considering the force exerted on superparamagnetic nanoparticles exposed to a spatially varying magnetic field. For a nanoparticle of volume $V_{p}$ and magnetic susceptibility $\chi_{p}$, suspended in a medium of susceptibility $\chi_{m}$, the magnetic force in a non-uniform magnetic field B(r) is given by

(3) $$F_{mag}(r)=\frac{V_{p}(\chi_{p}-\chi_{m})}{\mu_{0}}\;B(r)\;\frac{dB}{dr},$$

where $\mu_{0}$ denotes the magnetic permeability of free space. This expression captures the tendency of magnetically susceptible particles to migrate toward regions of higher magnetic field intensity.

At the length and time scales relevant for nanoscale drug carriers, inertial effects are negligible and particle motion occurs in the low-Reynolds-number regime. Under these conditions, the magnetic force is balanced by viscous drag, resulting in a constant magnetic drift velocity for a given local magnetic field and field gradient. The resulting drift velocity can be expressed as

(4) $$v_{mag}(r)=\alpha \;B(r)\;\frac{dB}{dr},\;\;\;\alpha =\frac{V_{p}(\chi_{p}-\chi_{m})}{6\pi \eta r_{p}\mu_{0}},$$

where η is the dynamic viscosity of the surrounding medium and $r_{p}$ is the nanoparticle radius. This formulation highlights that magnetic targeting introduces a directed transport mechanism proportional to both the magnetic field strength and its spatial gradient. The magnetic drift velocity $v_{\mathrm{mag}}(r)$ is a central component of the model, describing the directed advection of drug carriers toward the cell membrane. It should be emphasized that the present model does not assume a globally spherically symmetric magnetic field. Instead, the magnetic drift term represents a local effective approximation of the magnetic field gradient projected along the radial direction. This approximation is consistent with experimentally realizable configurations in which the magnetic field can be locally approximated as varying linearly over the cellular length scale, so that the magnetic field gradient is treated as approximately constant within the modeled domain. Consequently, the formulation captures the relevant transport physics without requiring a full electromagnetic field solution. All parameters required to compute $v_{\mathrm{mag}}(r)$ are summarized in Table 3. These include nanoparticle radius $r_{p}$, magnetic susceptibility of the particle $\chi_{p}$, magnetic susceptibility of the medium $\chi_{m}$, dynamic viscosity of the extracellular medium η, magnetic field strength B(r) and its spatial gradient $\frac{dB}{dr}$.

**Figure 7:**
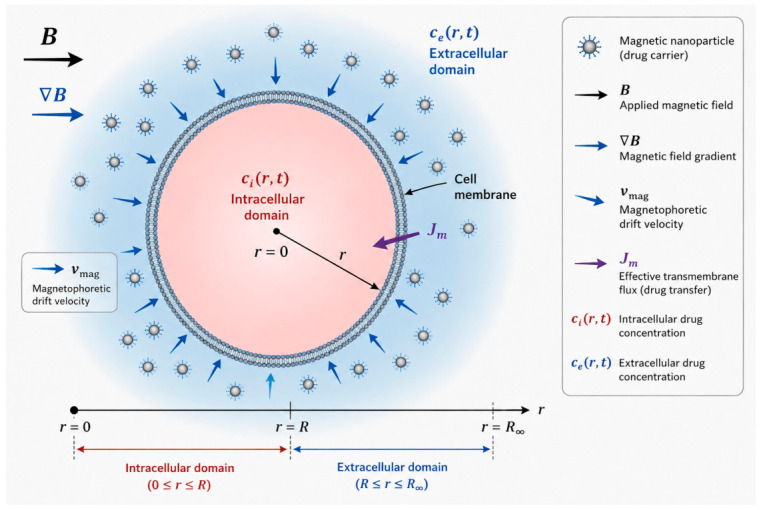
Schematic representation of the model geometry and transport mechanisms, including $c_{e}(r,t)$, $c_{i}(r,t)$, $v_{mag}$, and the effective transmembrane flux $J_{m}$ across the cell membrane.

The geometrical and transport assumptions of the model are summarized schematically in Figure 7. The figure shows the spherical cell of radius R embedded in an extracellular domain extending to $R_{\infty }$, together with the intracellular and extracellular concentration fields $c_{i}(r,t)$ and $c_{e}(r,t)$. It also highlights the applied magnetic field B, the magnetic field gradient ∇B, the corresponding magnetophoretic drift velocity $v_{mag}$, and the effective transmembrane flux $J_{m}$ across the cell membrane.

In the present model, the quantities entering Equation (4) should be understood as effective carrier parameters. In particular, $r_{p}$ represents the effective hydrodynamic radius of the drug-loaded magnetic carrier rather than only the bare magnetic core radius, while $\chi_{p}$ denotes an effective magnetic susceptibility that depends on the core composition, magnetic loading, coating, and possible clustering state [16,22]. The Fe content of the ${Fe}_{3}O_{4}$-based carrier is not determined from a specific synthesis protocol in the present theoretical study. Instead, the magnetic material loading is represented implicitly through the effective magnetic susceptibility $\chi_{p}$ used in the magnetophoretic force expression. For an ideal stoichiometric ${Fe}_{3}O_{4}$ core, the Fe/O ratio is fixed by the chemical formula; however, in real carriers, the effective magnetic response may vary with synthesis conditions, oxidation state, core size, coating thickness, aggregation, and the fraction of magnetic material within the drug carrier. Therefore, $\chi_{p}$ should be interpreted as an effective parameter that incorporates the net magnetic contribution of the ${Fe}_{3}O_{4}$ phase rather than as a direct calculation of Fe content. Surface functionalization and aggregation can therefore influence the magnetic drift velocity through changes in hydrodynamic drag, effective particle size, and magnetic susceptibility [23,24]. These effects are not modeled explicitly at the particle-interaction level, but they are incorporated phenomenologically through the effective parameters used to calculate $v_{\mathrm{mag}}$.

The model further assumes a dilute suspension of magnetic carriers, so that particle–particle interactions are neglected in the governing transport equations. This approximation is appropriate when the mean interparticle distance is sufficiently large and when electrostatic or steric stabilization provided by surface coatings reduces aggregation and maintains colloidal stability [16,23]. Under these conditions, the dominant directed contribution to particle motion is the externally imposed magnetophoretic drift, whereas magnetic dipole–dipole and electrostatic interactions between individual carriers are treated as secondary effects [22,36]. Their influence is therefore incorporated only implicitly through effective quantities such as the hydrodynamic radius, diffusion coefficient, and magnetic susceptibility.

For comparison, clinically used MRI systems typically operate at magnetic field strengths of 1.5–3 T, with research systems reaching up to 7 T, while magnetic field gradients are generally in the range of 30–80 mT/m [44]. In contrast, significantly higher gradients can be achieved in localized magnetic targeting configurations, such as micro-magnets or near-field setups, where gradients may reach values on the order of tens to hundreds of T/m [22].

The dynamic viscosity η plays a central role in determining the efficiency of magnetic targeting. According to Equation (4), the magnetic drift velocity is inversely proportional to the viscosity of the surrounding medium; therefore, an increase in η reduces magnetophoretic mobility, whereas lower viscosity enhances magnetically induced transport. In the present simulations, viscosity is included explicitly through the drift-velocity coefficient used to calculate $v_{mag}$. For the baseline simulations, the extracellular dynamic viscosity is taken as $\eta_{e}=1.0\times 10^{-3}\;Pa\;s$, corresponding to a water-like physiological environment at body temperature. To account for increased resistance in denser extracellular environments, higher effective viscosities in the range $\eta_{e}=1–5\times 10^{-3}\;Pa\;s$ are also considered.

Viscosity also affects the stability and transport behavior of nanoparticle carriers indirectly. Higher viscosity reduces Brownian mobility and magnetophoretic drift, thereby slowing redistribution of particles toward the membrane. In biological media, the effective viscosity is closely related to medium composition, protein adsorption, aggregation state, and hydrodynamic size of the carrier. These effects can modify colloidal stability and are represented in the present reduced-order model through effective transport parameters, including η, the hydrodynamic radius, the diffusion coefficient, and the magnetic susceptibility [16,23,38,39].

### 3.1. Extracellular Region: Diffusion and Magnetic Targeting

Drug transport in the extracellular region is modeled as a combined diffusion–advection process that accounts for magnetically induced drift of drug-loaded nanoparticles suspended in the extracellular fluid surrounding the cell. The extracellular drug flux is defined as the sum of a diffusive contribution and a magnetically induced advective contribution,

(5) $$J_{e}=-D_{e}\nabla c_{e}+v_{mag}(r)\;c_{e},$$

where $c_{e}(r,t)$ denotes the extracellular drug concentration and $D_{e}$ is the extracellular diffusion coefficient. The advective term arises from magnetic targeting and is governed by the magnetic drift velocity $v_{mag}(r)$, defined in Equation (4). This transport mechanism corresponds to magnetophoretic transport of nanoparticles in a magnetic field gradient, which is commonly referred to as magnetophoresis in the literature [22]. This term represents the directed motion of magnetically responsive carriers in the presence of a magnetic field gradient. Physically, magnetic drift biases nanoparticle transport toward the tumor cell membrane, resulting in increased drug accumulation in its immediate vicinity. Importantly, magnetic targeting does not directly alter membrane properties but instead enhances the extracellular concentration gradient at the membrane surface, thereby increasing the driving force for transmembrane diffusion.

Applying conservation of mass in the extracellular region leads to the governing advection–diffusion equation for the extracellular drug concentration,

(6) $$\frac{\partial c_{e}}{\partial t}=\frac{1}{r^{2}}\frac{\partial }{\partial r}\left[r^{2}\left(D_{e}\frac{\partial c_{e}}{\partial r}-v_{mag}(r)\;c_{e}\right)\right],R<r<R_{\infty }$$

This equation describes the coupled effects of passive diffusion and magnetically induced advection on drug transport in the extracellular domain.

The extracellular diffusion coefficient $D_{e}$ is a key parameter governing transport dynamic, as it determines the rate at which the drug spreads toward the cell membrane in the absence of magnetic effects. Its magnitude depends strongly on the size and physicochemical nature of the transported species. For magnetically targeted nanoscale drug carriers, such as polymeric nanoparticles or liposomal systems, diffusion coefficients in the range $10^{-13}$–$10^{-11}\;m^{2}/s$ are appropriate, reflecting their reduced mobility in the extracellular fluid. In contrast, free molecular drugs typically exhibit diffusion coefficients on the order of $10^{-9}\;m^{2}/s$ in aqueous environments, Table 4 [25,26].

### 3.2. Intracellular Region: Diffusion and Uptake

Within the tumor cell, drug transport is governed by intracellular diffusion and uptake processes. Under the assumption of spherical symmetry, the intracellular drug concentration $c_{i}(r,t)$ satisfies the following reaction–diffusion equation:

(7) $$\frac{\partial c_{i}}{\partial t}=\frac{1}{r^{2}}\frac{\partial }{\partial r}\left(r^{2}D_{i}\frac{\partial c_{i}}{\partial r}\right)-k_{u}\;c_{i},\;\;\;\;0<r<R,$$

where $D_{i}$ denotes the intracellular diffusion coefficient and $k_{u}$ represents the rate at which the drug is removed from the freely diffusing intracellular pool. No magnetic field–induced transport term is included in the intracellular governing equation. This modeling choice is motivated by both physical and biological considerations. First, intracellular transport is generally dominated by passive diffusion and biochemical interactions rather than by externally driven forces. Most nanoscale drug carriers either do not actively migrate within the cell or lose their direct motion upon internalization. As a result, magnetically induced drift is not expected to play a significant role in intracellular transport. Second, the effective species undergoing intracellular transport is assumed to be the drug released from its carrier rather than intact magnetic nanoparticles retaining strong magnetic responsiveness. Once inside the cell, drug molecules diffuse through the cytoplasm and interact with intracellular targets, while the contribution of magnetic forces becomes negligible. Finally, magnetic forces acting within the intracellular environment are expected to be strongly attenuated due to several factors, including effectively reduced magnetic responsiveness at the intracellular scale, the high effective viscosity of the cytoplasm, and strong spatial confinement caused by cytoskeletal structures and intracellular membranes. These effects collectively suppress magnetically driven motion at the intracellular scale. For these reasons, magnetic drift is considered relevant only in the extracellular region, where drug-loaded nanoparticles move freely in the surrounding fluid. This reduction in effective magnetic responsiveness is primarily due to the complex intracellular environment, characterized by high macromolecular crowding, increased effective viscosity, and strong steric constraints imposed by cytoskeletal structures and intracellular organelles. These factors significantly hinder the translational mobility of nanoparticle carriers and suppress directed motion under external forces [36]. In addition, nanoparticle internalization is typically followed by sequestration into endosomal and lysosomal compartments, further limiting their ability to respond to external magnetic field gradients [27]. As a result, magnetophoretic transport is expected to be negligible within the intracellular domain.

The uptake or binding rate $k_{u}$ accounts for irreversible or quasi-irreversible processes that remove the drug from the freely diffusing intracellular concentration. The parameter $k_{u}$ is therefore used as an effective first-order rate constant representing intracellular uptake, binding, retention, or removal from the freely diffusing drug pool. Such processes may include binding to intracellular proteins, metabolic degradation, or sequestration into cellular organelles. The value of $k_{u}$ is drug- and cell-type-specific and depends on experimental conditions. Typical values reported for tumor cells span several orders of magnitude. For small-molecule chemotherapeutic agents, such as doxorubicin or cisplatin, uptake or binding rates in the range $k_{u}∼10^{-4}–10^{-2}\;s^{-1}$ are commonly reported [45]. For nanoparticle-based or liposomal drug delivery systems, slower effective uptake rates are expected, with typical values in the range $k_{u}∼10^{-5}–10^{-3}\;s^{-1}$ [41].

### 3.3. Boundary and Initial Conditions

At the center of the spherical tumor cell (r=0), spherical symmetry implies a zero-radial gradient of the intracellular drug concentration:

(8) $${\frac{\partial c_{i}}{\partial r}|}_{r=0}=0.$$

This condition ensures that no unphysical flux arises at the cell center and preserves mass conservation within the intracellular domain.

The cell membrane is modeled as a semi-permeable interface that enforces continuity of mass flux between the extracellular and intracellular regions. The extracellular and intracellular fluxes at the membrane are defined as

(9) $$J_{e}(R^{+},t)=-D_{e}{\frac{\partial c_{e}}{\partial r}|}_{R^{+}}+v_{\mathrm{mag}}(R)\;c_{e}(R,t),$$

(10) $$J_{i}\left(R^{-},t\right)=-D_{i}{\frac{\partial c_{i}}{\partial r}|}_{R^{-}},$$

where $J_{e}$ and $J_{i}$ represent the extracellular and intracellular fluxes, respectively. The transmembrane flux is then expressed through the membrane permeability $P_{m}$ as

(11) $$J_{m}(t)=J_{e}(R^{+},t)=J_{i}(R^{-},t)=-P_{m}(c_{e}(R,t)-c_{i}(R,t)).$$

In this effective continuum formulation, drug transfer across the membrane is controlled by the extracellular concentration evaluated at the membrane surface, $c_{e}(R,t)$, rather than by a prescribed finite capture distance from the membrane. Nanoparticles located farther from the membrane influence drug delivery indirectly by contributing to the time-dependent extracellular concentration field through diffusion and magnetophoretic drift. Thus, only the near-membrane concentration enters the boundary flux explicitly, while transport from more distant extracellular regions is accounted for through the evolution of $c_{e}(R,t)$.

It should also be noted that the present model does not explicitly track the fate of the magnetic carrier after it reaches the cell membrane. The transported variable represents an effective drug concentration associated with magnetic carriers, while the membrane condition describes the net transfer of a drug into the intracellular domain. In this reduced-order formulation, magnetic carriers are assumed to remain in the extracellular/membrane-associated region after contributing to drug delivery, but their possible accumulation, retention, recycling, or steric blocking effects are not explicitly resolved. These effects are instead absorbed into the effective permeability parameter $P_{m}$ and the effective transport parameters used in the model.

The membrane transfer is therefore not assumed to be instantaneous. Instead, the finite permeability coefficient $P_{m}$ introduces a characteristic membrane-transfer time scale, which limits the rate at which a drug can enter the intracellular domain. When $P_{m}$ is small, the membrane acts as a kinetic barrier and the transmembrane flux remains limited even if the extracellular concentration near the membrane is high. Conversely, larger values of $P_{m}$ correspond to faster effective transfer, but the process is still governed by the finite flux condition rather than by instantaneous equilibration.

This interface condition ensures conservation of drug mass across the cell membrane by enforcing continuity of flux between the extracellular and intracellular domains. The extracellular and intracellular transport processes are coupled through the membrane permeability coefficient $P_{m}$, which governs the exchange of drugs between the two compartments. When $P_{m}$ is small, the membrane acts as the primary transport barrier and limits drug entry into the cell, resulting in a low transmembrane flux $J_{m}$ even in the presence of high extracellular drug concentrations. In contrast, when $P_{m}$ is large, transport across the membrane is rapid and the transmembrane flux is primarily determined by the concentration difference between the extracellular and intracellular regions, $c_{e}-c_{i}$. Magnetic targeting indirectly enhances intracellular uptake by increasing the extracellular drug concentration near the membrane, $c_{e}(R,t)$, which raises the transmembrane flux $J_{m}$. While $J_{e}$ and $J_{i}$ describe transport on either side of the membrane, only $J_{m}$ quantifies the effective drug delivery into the cell.

At the beginning of the simulation (t=0), the extracellular and intracellular drug concentrations are initialized as:

(12) $$c_{e}(r,0)=c_{0},\;\;c_{i}(r,0)=0.$$

$c_{0}$ represents the initial extracellular drug concentration and serves as a reference. It may be expressed in units of mol/m^3^, mg/L, or particle number density. Typical examples include small molecules in aqueous solution: $c_{0}=1\;\mathrm{mol}/m^{3}$ (∼1 mM), which serves as a reference concentration used for normalization of the transport equations and does not represent a specific physiological nanoparticle concentration, and nanoparticle-based drug carriers: $c_{0}$ expressed as particle number per unit volume. The intracellular domain is assumed to be initially free of the drug, reflecting that uptake occurs only through membrane transport over time. In numerical implementations, the extracellular domain up to the computational boundary $R_{\infty }$ is initialized with $c_{0}$, ensuring a uniform starting condition. Subsequent evolution of $c_{e}$ and $c_{i}$ is governed by diffusion, magnetic drift, and transmembrane flux. The intracellular concentration increases exclusively via transmembrane transport as the simulation progresses, driven by diffusion and magnetic drift in the extracellular region. Thus, the initial extracellular concentration $c_{0}$ is assumed to be spatially uniform throughout the domain at t=0. After initialization, diffusion and magnetic drift drive the redistribution of the drug toward the cell membrane, which in turn governs the subsequent transmembrane flux and the progressive accumulation of the drug inside the cell. The model results are governed by relative concentration gradients and dimensionless transport parameters, such that the absolute value of $c_{0}$ does not affect the qualitative conclusions of the study.

To ensure numerical reliability, the finite-difference scheme was tested for grid convergence and stability. Simulations were performed using progressively refined spatial grids, confirming that the solution remains unchanged within numerical tolerance. Additionally, limiting cases were verified: in the absence of magnetic drift ($v_{\mathrm{mag}}$ = 0), the model reduces to classical diffusion, reproducing expected concentration profiles. Mass conservation across the membrane interface was also confirmed throughout the simulations.

### 3.4. Dimensionless Formulation

To generalize the system and identify the dominant transport mechanisms, we introduce dimensionless variables, a dimensionless radial coordinate and dimensionless diffusion time:

(13) $$\tilde{r}=\frac{r}{R},\tilde{t}=\frac{D_{e}t}{R^{2}}.$$

Using these variables, the governing equations can be expressed in terms of dimensionless parameters that characterize the relative importance of advection, diffusion, and membrane transport.

Further, the magnetic Peclet number is defined as

(14) $$Pe_{mag}=\frac{v_{mag}(R)\;R}{D_{e}}$$

where $v_{\mathrm{mag}}(R)$ is the magnetic drift velocity at the cell surface. This number quantifies the relative contribution of magnetically induced advection to diffusive transport in the extracellular space: (a) $Pe_{\mathrm{mag}}\ll 1$: diffusion-dominated regime, where magnetic drift is negligible, (b) $Pe_{\mathrm{mag}}∼1$: mixed regime, where diffusion and magnetic drift contribute comparably, and (c) $Pe_{\mathrm{mag}}\gg 1$: drift-dominated regime, where magnetically induced advection governs transport toward the membrane.

The membrane Biot number is given by

(15) $$Bi=\frac{P_{m}R}{D_{e}},$$

where $P_{m}$ is the cell membrane permeability. This number measures the relative resistance of the membrane compared to diffusion in the extracellular medium: (a) Bi≪1: membrane-limited regime, with transmembrane flux controlled primarily by $P_{m}$, (b) Bi∼1: comparable contributions from membrane transport and extracellular diffusion, and (c) Bi≫1: diffusion-limited regime, where the membrane offers little resistance.

The ratio of intracellular to extracellular diffusivity is defined as

(16) $$\delta =\frac{D_{i}}{D_{e}}.$$

This parameter captures the relative mobility of the drug inside the cell compared to the extracellular space. Together, these dimensionless numbers provide a framework to classify transport regimes, assess the efficiency of magnetic targeting, and evaluate the limiting factors in drug delivery. By systematically varying $Pe_{\mathrm{mag}}$, Bi, and δ, one can identify optimal combinations of magnetic field gradients, nanoparticle properties, and membrane permeability to enhance intracellular drug uptake. The parameter $Pe_{mag}$ controls the relative strength of magnetically induced drift compared to diffusion and is primarily governed by the applied magnetic field gradient and nanoparticle magnetic properties. Increasing $Pe_{mag}$ enhances drug accumulation near the cell membrane, but only up to the point where membrane transport becomes rate-limiting. This limitation is captured by the Biot number Bi, which quantifies the resistance of the membrane relative to extracellular diffusion. For small Bi, improvements in magnetic targeting yield diminishing returns, as transmembrane transport constrains uptake. In contrast, larger values of Bi allow enhanced surface accumulation to translate directly into increased intracellular flux. The diffusivity ratio δ further modulates intracellular drug distribution by determining how efficiently the drug spreads once inside the cell. Together, these parameters define distinct transport regimes and provide a systematic framework for optimizing magnetic field gradients, nanoparticle size and susceptibility, and membrane permeability to maximize intracellular drug delivery.

### 3.5. Numerical Simulation of Coupled Diffusion–Drift Transport

The present framework enables a systematic numerical investigation of magnetically targeted drug delivery at the single-cell level. By solving the coupled extracellular and intracellular transport equations using a finite-difference scheme, the model yields the full spatiotemporal evolution of drug concentrations. This allows quantitative analysis of key delivery characteristics, including the enhancement of transmembrane drug flux induced by magnetic drift, the identification of critical magnetic field gradients required for effective targeting, and the sensitivity of intracellular drug uptake to membrane permeability $P_{m}$ and intracellular uptake kinetics $k_{u}$. Moreover, the framework captures transitions between diffusion-dominated and drift-enhanced transport regimes, providing insight into the conditions under which magnetic targeting becomes a dominant mechanism of drug delivery. The main outputs of the simulations are the spatiotemporal concentration fields, $c_{e}(r,t),$ and $c_{i}(r,t),$ which describe how the drug redistributes in the extracellular space and how much enters the cell over time. These fields form the basis for all derived quantities.

From the computed spatiotemporal concentration fields, several key derived quantities are extracted to quantify the efficiency of magnetically targeted drug delivery. The most important of these is the transmembrane drug flux, $J_{m}(t)$, defined by Equation (11), which directly measures the rate of drug entry into the cell and serves as a primary indicator of targeting performance. This quantity enables systematic comparison of drug uptake with and without an applied magnetic field, as well as evaluation of the dependence of cellular uptake on the magnetic field gradient ∇B. A complementary and biologically intuitive metric is the cumulative intracellular drug amount,

(17) $$M_{i}(t)=4\pi \int_{0}^{R}c_{i}(r,t)\;r^{2}\;dr,$$

which represents the total amount of a drug internalized by the cell over time. Magnetic targeting acts indirectly by increasing the extracellular drug concentration at the membrane surface, $c_{e}(R,t)$, which in turn enhances the transmembrane flux and promotes intracellular accumulation. To quantify the overall benefit of magnetic targeting, the targeting efficiency is defined as

(18) $$\eta_{M}(t;v_{mag})=\frac{M_{i}(t;v_{mag})}{M_{i}(t;0)},$$

where values of ${\;\eta }_{M}>1$ indicate enhanced intracellular delivery due to the applied magnetic field.

The numerical results highlight the fundamental mechanism of magnetic targeting: magnetic drift does not alter membrane permeability but instead increases the local drug concentration in the vicinity of the cell membrane, thereby strengthening the concentration gradient that drives transmembrane transport. At the same time, the model reveals intrinsic limitations of this strategy. When the membrane permeability $P_{m}$ is small, the membrane becomes the rate-limiting step regardless of extracellular accumulation, whereas for weak magnetic field gradients ∇B, magnetic drift is negligible and transport remains diffusion-dominated.

### 3.6. Numerical Solution via the Finite Difference Method

To obtain numerical solutions of the advection–diffusion Equation (9) and reaction–diffusion Equation (9), we employ the finite difference method (FDM), a classical and widely validated technique for partial differential equations [25,34,35]. The approach consists of discretizing both the spatial domain and the temporal domain, replacing continuous derivatives with discrete approximations that can be implemented on a computational grid. Let $c\left(r,t\right)$ denote a general concentration field, which may represent $c_{e}\left(r,t\right)$ or $c_{i}\left(r,t\right)$. We define a spatial grid with $N_{r}+1$, nodes, $r_{i}=i\;\Delta r$ for $i=0,1,\ldots ,N_{r}$, and a temporal grid $t_{j}=j\;\Delta t$ for $j=0,1,\ldots ,N_{t}$. The concentration at node i and time step j is denoted as $c_{i}^{j}\equiv c\left(r_{i},t_{j}\right)$.

In spherical coordinates, the diffusion operator involves both first- and second-order radial derivatives. At interior grid points $i=1,2,\ldots ,N_{r}-1$, these derivatives are approximated using second-order central differences. The first derivative is written as

(19) $${\left.\frac{\partial c}{\partial r}\right|}_{r_{i}}\approx \frac{c_{i+1}^{j}-c_{i-1}^{j}}{2\Delta r},$$

and the second derivative as

(20) $${\left.\frac{\partial^{2}c}{\partial r^{2}}\right|}_{r_{i}}\approx \frac{c_{i+1}^{j}-2c_{i}^{j}+c_{i-1}^{j}}{{\left(\Delta r\right)}^{2}}.$$

Substitution of these expressions into the governing equations yields a discrete representation of the radial diffusion operator that preserves flux conservation under spherical symmetry.

Time integration is performed using an explicit forward Euler scheme. The temporal derivative is approximated as

(21) $${\left.\frac{\partial c}{\partial t}\right|}_{r_{i},t_{j}}\approx \frac{c_{i}^{j+1}-c_{i}^{j}}{\Delta t}.$$

Combining the spatial and temporal discretizations, for the intracellular region, the explicit FDM scheme for internal nodes is:

(22) $$c_{i,i}^{\;j+1}=c_{i,i}^{\;j}+\Delta t\left[D_{i}\left(\frac{c_{i,i+1}^{\;j}-2c_{i,i}^{\;j}+c_{i,i-1}^{\;j}}{\Delta r^{2}}+\frac{2}{r_{i}}\cdot \frac{c_{i,i+1}^{\;j}-c_{i,i-1}^{\;j}}{2\Delta r}\right)-k_{u}c_{i,i}^{\;j}\right]$$

and

(23) $$c_{i,0}^{\;j+1}=c_{i,0}^{\;j}+\Delta t\left[D_{i}\left(\frac{2(c_{i,1}^{\;j}-c_{i,0}^{\;j})}{\Delta r^{2}}\right)-k_{u}c_{i,0}^{\;j}\right],$$

for the boundary node at r=0. For the extracellular region, the FDM scheme for internal knots is

(24) $$c_{e,i}^{\;j+1}=c_{e,i}^{\;j}+\Delta t\left[D_{e}\left(\frac{c_{e,i+1}^{\;j}-2c_{e,i}^{\;j}+c_{e,i-1}^{\;j}}{\Delta r^{2}}+\frac{2}{r_{i}}\frac{c_{e,i+1}^{\;j}-c_{e,i-1}^{\;j}}{2\Delta r}\right)\;\;\;\;\;\;\;\;-v_{i}\;\frac{c_{e,i+1}^{\;j}-c_{e,i-1}^{\;j}}{2\Delta r}-\left(\frac{v_{i+1}-v_{i-1}}{2\Delta r}+\frac{2v_{i}}{r_{i}}\right)c_{e,i}^{\;j}\right],$$

where $v_{i}\equiv v_{mag}(r_{i})$. At the boundary $r_{i}=R$, the condition given by Equation (11) leads to expressions for the concentrations on the left and right sides of the boundary

(25) $$c_{i,N}^{\;j}=\frac{a\;c_{i,N-1}^{\;j}\;(P_{m}+b+v_{R})+P_{m}\;b\;c_{e,2}^{\;j}}{\Delta },$$

(26) $$c_{e,1}^{\;j}=\frac{(P_{m}+a)\;b\;c_{e,2}^{\;j}-P_{m}\;a\;c_{i,N-1}^{\;j}}{\Delta },$$

where $\Delta =(P_{m}+a)(P_{m}+b+v_{R})+P_{m}^{2}$ is an introduced parameter, $a\;=D_{i}/\Delta r,$ and $b\;=D_{e}/\Delta r$. For the explicit scheme, numerical stability is ensured by selecting the time step to satisfy the standard diffusion stability criterion

(27) $$\Delta t\leq \frac{{\left(\Delta r\right)}^{2}}{2D_{max}},$$

where $D_{max}$ is the maximum effective diffusion coefficient encountered in the simulations. Δr is chosen to have a hundred nodes in the cell interior, while Δt is chosen to satisfy Equation (27). Implicit or Crank–Nicolson schemes can also be applied to improve numerical stability, particularly for stiff reaction terms or fine spatial grids [25]. The FDM implementation naturally accommodates spherically symmetric boundary conditions, including the Neumann condition at the centre (r=0) and Robin or Dirichlet conditions at the cell membrane (r=R). To ensure numerical robustness, simulations were repeated for refined spatial and temporal resolutions, confirming convergence of the solution and conservation of total mass within numerical tolerance.

### 3.7. Parameterization of Magnetic Carriers and Model Systems

In this section, representative classes of magnetic nano- and microparticle carriers are introduced to define physically realistic parameter ranges used in the model, rather than to provide a comprehensive literature review. Magnetic nano and microparticles have been extensively investigated as drug carriers to improve targeting efficiency and reduce nonspecific distribution associated with conventional delivery systems [16,22]. By incorporating magnetic components, these carriers can be guided through the extracellular fluid surrounding tumor cells and concentrated near the target site using externally applied magnetic fields, thereby overcoming rapid clearance by the reticuloendothelial system and limited site specificity. Magnetic particles based on spinel ferrites, such as iron- or zinc-substituted iron oxides, exhibit strong magnetic responsiveness, enabling their manipulation within the vascular and interstitial environments and promoting retention in regions exposed to magnetic field gradients. Structural designs such as porous or hollow architectures further enhance their functionality by increasing surface area and providing internal cavities for high drug loading. Hybrid systems combining magnetic nanoparticles with biodegradable polymers have also been developed, offering favorable magnetic responsiveness together with controlled and stimulus-sensitive drug release. In particular, pH-responsive formulations have demonstrated rapid initial release followed by sustained delivery under tumor-relevant conditions. At larger length scales, nano-in-microparticle assemblies, in which magnetic nanoparticles are embedded within microparticles, have shown promise for magnetically guided delivery in vivo, enabling localized drug accumulation under external magnetic actuation. Collectively, these magnetic carrier systems provide a versatile platform for directing drug-loaded particles through the extracellular space toward the tumor cell membrane, forming the physical basis for magnetically enhanced transmembrane drug transport. Table 5 provides an overview of representative magnetic nano and microparticle carriers used in magnetically targeted drug delivery, together with their structural characteristics and magnetic behavior, which critically determine their response to externally applied magnetic fields. The magnetic behavior of iron-oxide-based carriers is strongly size-dependent, with superparamagnetic properties typically observed for single-domain cores below approximately 20–30 nm, while larger particles or clustered systems may exhibit ferrimagnetic or multi-domain behavior [16].

To enable a controlled and physically consistent comparison between healthy and tumor cells, two representative cellular parameter sets are introduced within the same continuum transport framework. All simulations are performed using identical cell geometry and identical external magnetic field conditions, such that any observed differences in intracellular drug accumulation arise exclusively from membrane- and uptake-related properties rather than from geometric effects or differences in magnetic forcing.

Both healthy and tumor cells are modeled as spherical cells with radius R=10 μm which is representative of typical mammalian cells and ensures identical surface-to-volume ratios and characteristic diffusion time scales in all simulations [46,47]. The extracellular and intracellular diffusion coefficients are treated as properties of the drug species and cytoplasmic environment rather than cell type. Accordingly, identical values are adopted for both healthy and tumor cells $D_{e}=1.0\times 10^{-11}\;m^{2}\;s^{-1},D_{i}=5.0\times 10^{-12}\;m^{2}\;s^{-1}.$ These values are consistent with reported diffusion coefficients for small molecules and nanoparticle-bound drugs in crowded extracellular environments and cytosolic media [48,49,50].

The principal biological distinction between healthy and tumor cells is introduced through the effective membrane permeability $P_{m}$, which represents the combined influence of lipid organization, membrane fluidity and heterogeneity, transporter-mediated pathways, and nanoscale membrane defects. Numerous experimental studies report increased membrane disorder and permeability in tumor cells compared to healthy counterparts, driven by altered lipid composition, cholesterol content, and disrupted bilayer asymmetry [51,52,53,54]. Based on these observations, the following representative values are adopted: healthy cell $P_{m}=1.0\times 10^{-8}\;m{\;s}^{-1},$ tumor cell, $P_{m}=1.0\times 10^{-7}\;m\;s^{-1}.$ This tenfold increase in effective membrane permeability provides a conservative yet physiologically plausible representation of tumor-associated membrane alterations within a continuum model [51,55].

Intracellular drug processing is represented by a first-order uptake/retention term with rate constant $K_{u}$, accounting phenomenologically for intracellular sequestration, binding, or metabolic trapping. Tumor cells are frequently characterized by enhanced intracellular retention of therapeutic agents due to altered organelle structure and dysregulated trafficking [45,56]. Accordingly, the following values are used for healthy cell $K_{u}=5.0\times 10^{-4}\;s^{-1}$, and for tumor cell: $K_{u}=1.0\times 10^{-3}\;s^{-1}$.

Magnetic forcing is prescribed through a fixed static magnetic field magnitude and gradient, $B_{0}=10\;T$, $\nabla B=100\;T\;m^{-1}$ representing a moderate yet experimentally accessible regime relevant to localized magnetic targeting systems and high-gradient experimental configurations, rather than standard MRI systems [22,57]. The magnetic drift velocity is computed from Equation (4) using these field parameters and the magnetic properties of the drug carrier. For the chosen values of $B_{0}$ and ∇B, the resulting drift velocity is $v_{mag}=5.0\times 10^{-7}\;m\;s^{-1}.$ This drift velocity is applied identically to healthy and tumor cells, reflecting the fact that magnetic drift is determined by the applied field and carrier properties rather than by intrinsic cellular characteristics [57,58].

For each cell type, two simulations are performed:

1. No-field case: $v_{mag}=0$, serving as the baseline reference.
2. Magnetic targeting case: $v_{mag}=5.0\times 10^{-7}\;m\;s^{-1}$, corresponding to the fixed $\left(B_{0},\nabla B\right)$.

All remaining parameters are kept unchanged between the two cases.

Flux-based analysis by introducing the total intracellular drug amount, given by Equation (17), which aggregates the spatially distributed intracellular concentration into a single physically interpretable metric (amount per cell). This quantity is especially convenient for comparing delivery efficiency across different magnetic forcing conditions, since it captures the combined effect of (i) transmembrane transport, (ii) slower intracellular spreading, and (iii) uptake-driven depletion.

A direct differential balance for $M_{i}(t)$ is obtained by integrating the intracellular transport equation over the spherical intracellular volume. Using the divergence theorem and the membrane flux as the boundary inflow, one obtains

(28) $$\frac{dM_{i}}{dt}=4\pi R^{2}\;J_{m}(t)-K_{u}\;M_{i}(t),{\;\;M}_{i}(0)=0$$

where $4\pi R^{2}\;J_{m}(t)$ is the instantaneous molar inflow rate through the membrane (surface area times flux density) and $K_{u}M_{i}(t)$ is the first-order intracellular uptake/removal rate. This relationship clarifies the complementary roles of flux and intracellular amount: $J_{m}(t)$ governs how rapidly a drug enters the cell, whereas $M_{i}(t)$ reflects how much free drug is retained inside at any time after accounting for uptake. In the limiting case $K_{u}=0$, $M_{i}(t)$ reduces to the time integral of the inflow rate, and flux-based and mass-based metrics become equivalent. When $K_{u}>0$, however, the same inflow history can lead to substantially different retained intracellular amounts depending on the balance between delivery and uptake.

When comparing magnetically targeted drug transport between healthy and tumor cells, special care must be taken in interpreting intracellular concentration levels. In the present model, healthy and tumor cells differ primarily in their intracellular uptake rate $k_{u}$, which represents irreversible or quasi-irreversible drug binding, sequestration, or metabolic trapping. Tumor cells are characterized by larger values of $k_{u}$, reflecting enhanced intracellular retention and processing of therapeutic agents. As a direct consequence, the instantaneous intracellular free-drug concentration $c_{i}(r,t)$, or its spatial integral $N_{i}(t)$, does not provide an unambiguous measure of delivery efficiency when uptake rates differ between cell types. A larger uptake rate reduces the free intracellular pool even when membrane delivery is enhanced, potentially masking the true impact of magnetic targeting. Therefore, intracellular concentration alone cannot be used as the sole metric for assessing targeting performance in systems with heterogeneous uptake kinetics. To resolve this ambiguity, we explicitly separate drug delivery across the membrane from intracellular drug fate by introducing two complementary cumulative quantities.

### 3.8. Cumulative Membrane Delivery

The total amount of a drug delivered into the cell through the membrane up to time t is defined as

(29) $$M_{in}\left(t\right)=\int_{0}^{t}4\pi R^{2}\;J_{m}\left(\tau \right)\;d\tau ,$$

where $J_{m}(t)$ is the transmembrane flux density. This quantity measures the cumulative inflow of a drug into the cell, independent of subsequent intracellular binding or removal processes. As such, $M_{in}(t)$ provides a direct and uptake-independent measure of membrane-level delivery and is ideally suited for quantifying the effect of magnetic targeting on transmembrane transport. Because magnetic fields act exclusively by enhancing extracellular transport and near-membrane accumulation, their primary influence is reflected in $M_{in}(t)$. This metric therefore isolates the physical contribution of magnetic drift from biological differences in intracellular processing.

### 3.9. Cumulative Intracellular Uptake

To quantify the biologically effective intracellular processing of the drug, we additionally define the cumulative uptake

(30) $$M_{upt}(t)=\int_{0}^{t}k_{u}\;M_{i}(\tau )\;d\tau$$

where $M_{i}(t)\;$ is the total amount of intercellular drugs defined by Equation (17). $M_{upt}(t)$ represents the total amount of the drug irreversibly taken up, bound, or retained inside the cell over time. Unlike $M_{in}(t)$, this quantity explicitly depends on the uptake rate $k_{u}$ and therefore captures intrinsic biological differences between healthy and tumor cells. While magnetic targeting influences $M_{upt}(t)$ indirectly, by increasing membrane delivery, this metric provides a more direct proxy for therapeutic effectiveness, as it reflects the cumulative intracellular drug processing rather than transient free-drug levels.

Within this framework, membrane delivery and intracellular uptake play distinct and complementary roles:

- $M_{in}(t)$ quantifies how much of the drug enters the cell, serving as a clean measure of magnetic targeting efficiency at the transport level.
- $M_{upt}(t)$ quantifies how much of the drug is effectively processed or retained, serving as a biologically relevant endpoint.
- The instantaneous intracellular amount $M_{i}(t)$ reflects the balance between delivery and uptake but should not be interpreted in isolation when uptake rates differ.

By analyzing both cumulative quantities, we avoid misleading conclusions that could arise from comparing free intracellular concentrations alone. This separation allows magnetic targeting effects to be evaluated independently of intracellular kinetics, while still enabling assessment of the overall therapeutic impact.

To quantify the relative impact of magnetic targeting under fixed magnetic conditions, the magnetic enhancement factor for each cell type X∈{healthy,tumor} is defined as

(31) $$E_{x,\mathrm{in}}\left(t\right)=\frac{M_{x,\mathrm{in}}^{B}\left(t\right)}{M_{x,\mathrm{in}}^{0}\left(t\right)},E_{x,\mathrm{upt}}\left(t\right)=\frac{M_{x,\mathrm{upt}}^{B}\left(t\right)}{M_{x,\mathrm{upt}}^{0}\left(t\right)}.$$

The relative therapeutic benefit of magnetic targeting is then defined as

(32) $$\Gamma_{\mathrm{in}}=\frac{E_{\mathrm{in}}^{\mathrm{tum}}}{E_{\mathrm{in}}^{\mathrm{healthy}}},\Gamma_{\mathrm{upt}}=\frac{E_{\mathrm{upt}}^{\mathrm{tum}}}{E_{\mathrm{upt}}^{\mathrm{healthy}}}\;.$$

Values Γ>1 indicate that magnetic targeting produces a larger fractional increase in intracellular drug accumulation in tumor cells than in healthy cells under identical magnetic field conditions.

## 4. Conclusions

In this work, we developed a theoretical framework for magnetically assisted drug transport across a tumor cell membrane at the single-cell level. The model combines extracellular diffusion and magnetically induced drift with membrane-limited transport and intracellular uptake, providing a consistent description of the coupled processes governing drug delivery.

The results show that magnetic field gradients enhance drug accumulation in the vicinity of the cell membrane, which in turn increases the transmembrane flux without modifying intrinsic membrane properties. This leads to a transport amplification effect, whereby magnetic targeting enhances existing biological differences, resulting in a more pronounced benefit for tumor cells compared to healthy cells.

By explicitly separating cumulative membrane delivery from cumulative intracellular uptake, the model resolves ambiguities associated with heterogeneous uptake kinetics and enables a clearer interpretation of delivery efficiency under magnetic forcing. This distinction provides a more robust basis for evaluating the role of magnetic targeting at the cellular level.

A dimensionless analysis identifies the magnetic Peclet number and the membrane Biot number as key parameters governing the transition between diffusion-dominated and drift-enhanced transport regimes. These parameters offer a systematic framework for optimizing magnetic field conditions and transport properties to improve intracellular drug delivery.

Overall, the proposed model provides a physically consistent and computationally efficient tool for analyzing magnetically targeted drug delivery. It establishes a foundation for future extensions toward more detailed descriptions of nanoparticle transport, cellular uptake mechanisms, and complex biological environments.

## Figures and Tables

**Table 1:** Parameters for PDE Simulation: Magnetically Targeted Drug Delivery.

Parameter	Symbol	Typical Value	Unit	Comment
Cell radius	R	$10\times 10^{-6}$	m	Typical mammalian cell
Extracellular domain radius	$R_{\infty }$	$200\times 10^{-6}$	m	Computational boundary
Extracellular diffusion coefficient	$D_{e}$	$1\times 10^{-12}–5\times 10^{-11}$	m^2^/s	For nanoparticles 50–100 nm
Intracellular diffusion coefficient	$D_{i}$	$1\times 10^{-13}–1\times 10^{-12}$	m^2^/s	Slower diffusion inside cytoplasm
Membrane permeability	$P_{m}$	$1\times 10^{-7}–1\times 10^{-6}$	m/s	Semi-permeable cell membrane
Uptake rate	$k_{u}$	$1\times 10^{-4}–1\times 10^{-3}$	s^−1^	Binding or metabolism rate
Nanoparticle radius	$r_{p}$	5–50	nm	Half of particle diameter
Particle susceptibility	$\chi_{p}$	0.1–1	–	Superparamagnetic Fe_3_O_4_
Medium susceptibility	$\chi_{m}$	−9 × 10^−6^	–	Water (diamagnetic)
Magnetic permeability	$\mu_{0}$	$4\pi \times 10^{-7}$	H/m	Vacuum
Magnetic field magnitude	B	0.1–2	T	Field strengths relevant to clinical/research MRI and magnetic targeting setups; gradients are treated separately
Magnetic field gradient	dB/dr	10–1000	T/m	Experimental targeting
Magnetic drift velocity	$v_{\mathrm{mag}}$	~10^−6^	m/s	μm/s scale drift
Extracellular concentration	$c_{0}$	1	mol/m^3^	Reference concentration

**Table 2:** Growth rate $S(v_{mag})$ extracted from linear fits of $\eta_{M}(t)$.

$v_{mag}$ (m s^−1^)	$S(v_{mag})$ (s^−1^)
$1\times 10^{-8}$	$4.0\times 10^{-4}$
$5\times 10^{-8}$	$2.4\times 10^{-3}$
$1\times 10^{-7}$	$5.0\times 10^{-3}$
$5\times 10^{-7}$	$4.3\times 10^{-2}$

**Table 3:** Required Parameters for $v_{\mathrm{mag}}\left(r\right)$.

Parameter	Typical Value	
$d=2r_{p}$	10–100 nm	Effective hydrodynamic diameter of coated or drug-loaded magnetic carriers. The magnetic core itself is typically smaller (≈10–30 nm) to ensure superparamagnetic behavior, while larger effective sizes arise due to surface coatings, aggregation, or carrier formulation.
$\chi_{p}$	0.1–1 (SI, dimensionless)	Effective susceptibility depending on iron-oxide phase, core size, magnetic loading, coating, and aggregation state.
$\chi_{m}$	$-9\times 10^{-6}$	Water, approximately diamagnetic
$\mu_{0}$	$4\pi \times 10^{-7}\;H/m$	Vacuum permeability
B(r)	0.1–2 T	Includes both clinical MRI field strengths (~1.5–3 T) and lower-field experimental or targeting configurations.
dB/dr	10–1000 T/m	Values representative of localized magnetic targeting systems; significantly higher than clinical MRI gradients (~30–80 mT/m).

**Table 4:** Typical values of $D_{e}$.

Particle/Drug Type	Typical $D_{e}$ Value	Comments
Small molecules (e.g., doxorubicin)	$5\times 10^{-10}–1\times 10^{-9}\;m^{2}/s$	Representative range for freely diffusing small molecular drugs in aqueous or dilute physiological media.
Liposomes/nanosphere carriers (50–100 nm)	$1\times 10^{-12}–5\times 10^{-11}\;m^{2}/s$	Lower apparent diffusivity resulting from increased hydrodynamic size and carrier-mediated transport.
Polymeric nanoparticles (100–200 nm)	$1\times 10^{-13}–1\times 10^{-12}\;m^{2}/s$	Strongly reduced mobility associated with larger carrier size and enhanced viscous resistance in the extracellular environment.

**Table 5:** Magnetic nano- and microparticles used in magnetic drug delivery and their magnetic properties [33].

Carrier Type	Typical Composition/Structure	Size Scale	Magnetic Behavior	Relevance for Magnetic Targeting
Spinel ferrite nanoparticles (MxFe_3−x_O_4_, M = Fe, Zn, Mn)	Solid, porous, or hollow iron-oxide–based nanoparticles	10–100 nm	Superparamagnetic (for core sizes below ~20–30 nm) or ferrimagnetic (for larger or clustered structures)	Strong response to magnetic field gradients; widely used for magnetic guidance and accumulation
Iron oxide nanoparticles (Fe_3_O_4_, γ-Fe_2_O_3_)	Single-core or clustered nanoparticles, often surface-functionalized	5–50 nm	Superparamagnetic	High magnetic susceptibility with negligible remanence; minimal aggregation after field removal
Core–shell magnetic nanoparticles	Magnetic core with polymer, silica, or inorganic shell	20–200 nm	Superparamagnetic	Enhanced stability, controlled drug loading and release, tunable magnetic response
Polymer–magnetic nanoparticle composite microspheres	Biodegradable polymer matrix embedding magnetic nanoparticles	1–10 μm	Superparamagnetic (effective)	High drug loading capacity and rapid magnetic response in extracellular fluids
Hollow or mesoporous magnetic particles	Mesoporous shell with internal cavity for drug encapsulation	50–500 nm	Typically superparamagnetic at the core level, but may exhibit effective ferrimagnetic behavior due to clustering or multi-domain structure	Large surface area and cavity volume enable efficient loading and magnetically assisted release
Nano-in-microparticles (NIMs)	Microparticles containing clusters of magnetic nanoparticles	1–5 μm	Superparamagnetic (collective response)	Improved magnetic retention and localized delivery under external magnetic fields
Magnetic microbubbles/microcapsules	Gas-filled or layered microstructures incorporating magnetic nanoparticles	1–10 μm	Superparamagnetic (due to embedded NPs)	Combined magnetic targeting and triggered release mechanisms

## Data Availability

The datasets generated and/or analyzed during the current study are available from the corresponding author on reasonable request.
